# Diversity and Prevalence of Clostridium innocuum in the Human Gut Microbiota

**DOI:** 10.1128/msphere.00569-22

**Published:** 2022-12-21

**Authors:** Disha Bhattacharjee, Clara Flores, Christine Woelfel-Monsivais, Anna M. Seekatz

**Affiliations:** a Department of Biological Sciences, Clemson University, Clemson, South Carolina, USA; Baylor College of Medicine

**Keywords:** *Clostridium innocuum*, genomics, metabolism, virulence, growth assay, gut microbiota, human microbiome

## Abstract

*Clostridia* are a polyphyletic group of Gram-positive, spore-forming anaerobes in the *Firmicutes* phylum that significantly impact metabolism and functioning of the human gastrointestinal tract. Recently, *Clostridia* were divided into two separate classes, *Clostridia* and *Erysipelotrichia*, based on phenotypic and 16S rRNA gene-based differences. While *Clostridia* include many well-known pathogenic bacteria, *Erysipelotrichia* remain relatively uncharacterized, particularly regarding their role as a pathogen versus commensal. Despite wide recognition as a commensal, the erysipelotrichial species Clostridium innocuum has recently been associated with various disease states. To further understand the ecological and potential virulent role of C. innocuum, we conducted a genomic comparison across 38 C. innocuum isolates and 194 publicly available genomes. Based on colony morphology, we isolated multiple C. innocuum cultivars from the feces of healthy human volunteers (*n* = 5). Comparison of the 16S rRNA gene of our isolates against publicly available microbiota data sets in healthy individuals suggests a high prevalence of C. innocuum across the human population (>80%). Analysis of single nucleotide polymorphisms (SNPs) across core genes and average nucleotide identify (ANI) revealed the presence of four clades among all available genomes (*n* = 232 total). Investigation of carbohydrate and protein utilization pathways, including comparison against the carbohydrate-activating enzyme (CAZyme) database, demonstrated inter- and intraclade differences that were further substantiated *in vitro*. Collectively, these data indicate genetic variance within the C. innocuum species that may help clarify its role in human disease and health.

**IMPORTANCE** *Clostridia* are a group of medically important anaerobes as both commensals and pathogens. Recently, a new class of *Erysipelotrichia* containing a number of reassigned clostridial species has emerged, including Clostridium innocuum. Recent studies have implicated C. innocuum as a potential causative agent of diarrhea in patients from whom Clostridioides difficile could not be isolated. Using genomic and *in vitro* comparison, this study sought to characterize C. innocuum in the healthy human gut. Our analyses suggest that C. innocuum is a highly prevalent and diverse species, demonstrating clade-specific differences in metabolism and potential virulence. Collectively, this study is the first investigation into a broader description of C. innocuum as a human gut inhabitant.

## INTRODUCTION

Commensal bacteria, viruses, fungi, and protozoa, collectively termed microbiota, dominate all surfaces of multicellular hosts. The collective genes provided by individuals or groups of microbes maintain health of the host, providing functions such as colonization resistance against pathogens via multiple mechanisms, including nutrient niche exclusion (1–3), modulation of oxygen or pH gradients along the gut (4, 5), and production of metabolites that harm pathogens (6, 7). However, variability of the gut microbiota across individual hosts (8, 9) and lack of characterization of many common gut inhabitants (10) complicate discernible conclusions about many individual members. Recent genomic and phenotypic studies have highlighted strain-level diversity within prominent gut species that can extricate our understanding of the microbiota in health (11–13), which is not captured by 16S rRNA gene-based surveys.

The human gut microbiota is predominantly occupied by anaerobic bacteria, with the most abundant phyla being *Firmicutes* and *Bacteroidetes* (14). The diversity and function of several prevalent *Bacteroidetes* species have been extensively investigated (15), leading to their use as prominent model organisms to understand gut microbiota function (16). For example, species within the *Bacteroides* genus are known to harbor hundreds of polysaccharide utilization loci (PULs) that degrade different glycans and carbohydrates (16), ultimately supplying nutrients to both the host and surrounding microbes that contribute to protection from pathogens. Many prevalent taxa within the polyphyletic, Gram-positive *Firmicutes* phylum remain more nebulous. Within the gut, the *Firmicutes* phylum is comprised of three classes based on analyses of 16S rRNA nucleotide sequence, *Bacilli*, *Clostridia*, and *Erysipelotrichia* (17–19). *Bacilli* and *Clostridia* comprise well-studied members with major implications in industrial applications, health, and disease (20–23). *Clostridia* as a group have been demonstrated to induce beneficial immune responses, in part via their ability to produce short-chain fatty acids that can attenuate gut inflammation (24, 25). In comparison, the importance of *Erysipelotrichia* in the human gut microbiota remains relatively unexplored. *Erysipelotrichia* include species that share a genomic resemblance to *Mollicutes*, a class of parasitic bacteria that are characterized by their distinct lack of cell walls compared to the phylum *Tenericutes* (26). As a group, *Erysipelotrichia* in the gut have been associated with host lipid metabolism (27, 28) and disease in humans (29). In mice, expansion of *Erysipelotrichia* species has been observed following antibiotic treatment (30) or when fed a Western diet (31).

The role of the erysipelotrichial species Clostridium innocuum in human health remains especially ambiguous. C. innocuum was first isolated from an appendiceal abscess but was deemed innocuous due to a lack of virulence observed in mice and guinea pigs (32). Recently reclassified from its original clostridial designation (33), C. innocuum has been identified as part of the “normal” gut microbiota via its capability to biodegrade glucose ureide (34). Although initial phenotypic description of the organism suggests a nonmotile, nonvirulent nature of C. innocuum (32), current literature suggests otherwise. It has been implicated in extraintestinal infection and Clostridioides difficile-like antibiotic-associated diarrhea (34–36), as well as in case studies of bacteremia, endocarditis, osteomyelitis, and peritonitis (27, 37–39). Most recently, a study on Crohn’s disease (CD) conducted in mice identified C. innocuum in inflamed intestinal tissue of patients with CD (40). Despite these studies, a direct virulence mechanism has yet to be identified (36, 40–42).

Given the putative prevalence of C. innocuum in the human gut microbiota, we aimed to investigate the genomic and phenotypic diversity of C. innocuum. We compared genomic phylogeny, functional capacity, and virulence factors across single isolates and publicly available genomes. Using a custom 16S rRNA database, we identified C. innocuum as a highly prevalent human gut inhabitant. Single nucleotide polymorphisms in core genes suggest that the C. innocuum species splits into multiple clades, characterized by differences in metabolism. While comparison to known virulence factors did not identify a direct link to previously associated disease studies, we did identify clade-specific putative virulence factors. Together, these data support a role for C. innocuum and related erysipelotrichial species in modulating the gut nutrient landscape, as well as a strain-specific role for potential virulence.

## RESULTS

### Clostridium innocuum is a prevalent human gut microbe.

**FIG 1:**
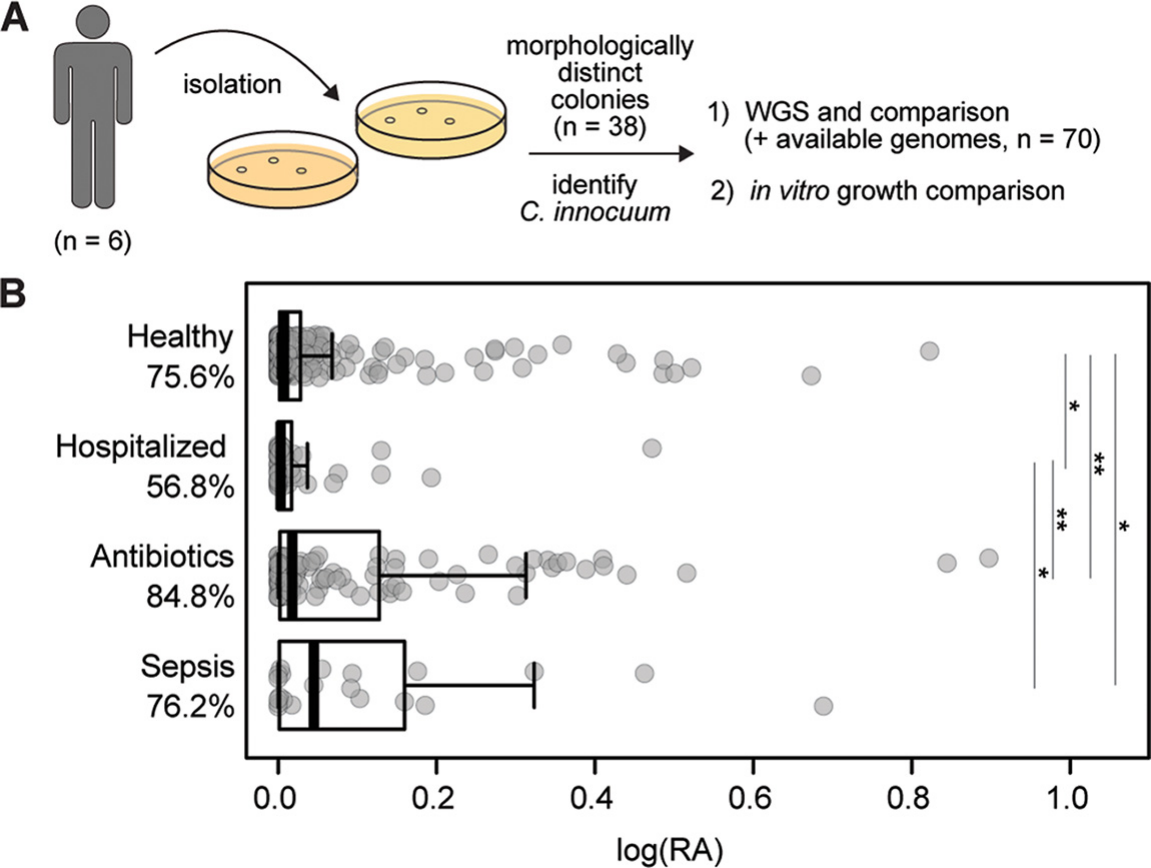
C. innocuum is a prevalent human gut bacterium. (A) Isolation pipeline design for C. innocuum. (B) Detection of C. innocuum in human feces across previously published 16S rRNA data sets using a custom classifier, categorized by published disease status. Log_10_ of relative abundance is displayed on the *x* axis, with prevalence (percent detected based on presence or absence). Pairwise Wilcoxon rank-sum test; *, *P* < 0.05; **, *P* < 0.005.

We screened five fresh fecal samples for the presence of C. innocuum strains as part of a larger study focused on cultivation of gut commensal bacteria (Fig. 1A). Sanger sequencing of the full-length 16S rRNA gene from morphologically distinct colonies confirmed the presence of 38 isolates that matched C. innocuum (80% similarity) (see Table S1 in the supplemental material), with each fecal sample yielding at least three distinct colonies. While metagenomic and 16S rRNA gene-based surveys suggest C. innocuum may be a common resident of the human gut microbiota, its prevalence across the human population is unknown. To broadly identify the presence of C. innocuum within the human gut microbiota, we compared multiple available 16S rRNA data sets from previous human gut microbiota studies to a curated database consisting of the 16S rRNA gene from our isolates (43, 44). Within these data sets, approximately 80% of samples (*n* = 420) contained C. innocuum sequences, suggesting a high prevalence of C. innocuum within the human gut microbiota (Fig. 1B). Although the relative abundance (RA) of C. innocuum in feces retrieved from 16S rRNA gene-based sequencing data was relatively low across all samples (mean RA = 0.22%), samples collected from patients on antibiotics (mean RA = 0.40%; *n* = 96) or with sepsis (mean RA = 0.46%; *n* = 24) were significantly increased compared to healthy controls (mean RA = 0.16%; *n* = 250).

10.1128/msphere.00569-22.2 TABLE S1 Excel sheet containing genome metadata. Download Table S1, XLSX file, 0.05 MB. Copyright © 2023 Bhattacharjee et al. 2023 Bhattacharjee et al. https://creativecommons.org/licenses/by/4.0/ This content is distributed under the terms of the Creative Commons Attribution 4.0 International license.

### Whole-genome comparison reveals four C. innocuum clades.

We next sought to identify genomic heterogeneity among all available C. innocuum genomes, including isolates within the current study (*n* = 38), genomes available on the Genome Taxonomy Database (GTDB) (*n* = 40) (40), genomes sequenced in a previously published study associated with Crohn’s disease (*n* = 31) (45), a newly published data set from clinical samples (*n* = 119) (46), and four fully annotated genomes (C. innocuum strains 14501, I46, LC-LUMC, and 2959) (47–49) (Table S1). Isolates were sequenced using Illumina technology, assembled (average depth of coverage, 90×), and annotated using Prokka (50). The newly assembled full-length 16S rRNA gene from all 232 C. innocuum genomes was used for taxonomic identification using both NCBI BLAST and EZBioCloud databases. These comparisons, as well as the full genomic assembly compared against the GTDB database, confirmed all genomes as *Erysipelotrichaceae* species. An initial maximum-likelihood tree of the full-length 16S rRNA gene obtained from the whole-genome assemblies revealed that most species clustered under one branch (Fig. S1A), suggesting that the 16S rRNA gene may not be an appropriate proxy for distinguishing C. innocuum strains.

10.1128/msphere.00569-22.3 FIG S1 Maximum-likelihood trees of C. innocuum based on full-length 16S rRNA gene- and PhyloPhlAn-selected markers. (A) Maximum-likelihood tree of full-length 16S rRNA generated by RAxML, aligned in Clustal Omega (99). (B) Maximum-likelihood tree of C. innocuum genomes based on 400 selected markers generated using PhyloPhlAn (500 bootstraps). The C. difficile genomes (CD630 and R20291) were used as an outgroup for both. Download FIG S1, EPS file, 1.4 MB. Copyright © 2023 Bhattacharjee et al. 2023 Bhattacharjee et al. https://creativecommons.org/licenses/by/4.0/ This content is distributed under the terms of the Creative Commons Attribution 4.0 International license.

We next analyzed the pangenome from *de novo*-assembled whole genomes of all putative C. innocuum genomes available (*n* = 232) using Roary (95% blast percentage identity) (Table S1) (51). Assemblies of 232 unique genomes averaged 4.6 Mbp, close to the type strain C. innocuum ATCC 14501 at 4.7 Mbp (48). We observed an average of 4,400 protein-encoding genes by coding sequences (CDS) obtained from Prokka (50). The gene accumulation curve followed Heap’s law with γ = 0.3322 ± 0.06 (*R*^2^ = 94.68%) (Fig. S2A). Heap’s decay parameter, alpha, totaled less than one (alpha = 0.794), suggesting an open pangenome for C. innocuum. This was further supported by the distribution of gene abundance across the number of genomes (Fig. S2B), which demonstrated that the number of unique genes (524 genes) common to all 232 genomes was less than those observed in a single genome, indicating extensive gene transference within and outside the species.

10.1128/msphere.00569-22.4 FIG S2 The pangenome of C. innocuum and related species is open. (A) Total number of genes as a function of number of all included genomes within the four clades (*n* = 232). Yellow-colored solid line represents the average number of total genes from five subsamplings, with transparent yellow as error bars. The function follows Heap’s law (top left corner). The alpha is from Heap’s law estimate, run over 500 iterations using micropan in R (*P* < 2e-16). (B) Total number of unique genes as a function of number of genomes (*P* < 2e-16). Download FIG S2, EPS file, 1.4 MB. Copyright © 2023 Bhattacharjee et al. 2023 Bhattacharjee et al. https://creativecommons.org/licenses/by/4.0/ This content is distributed under the terms of the Creative Commons Attribution 4.0 International license.

**FIG 2:**
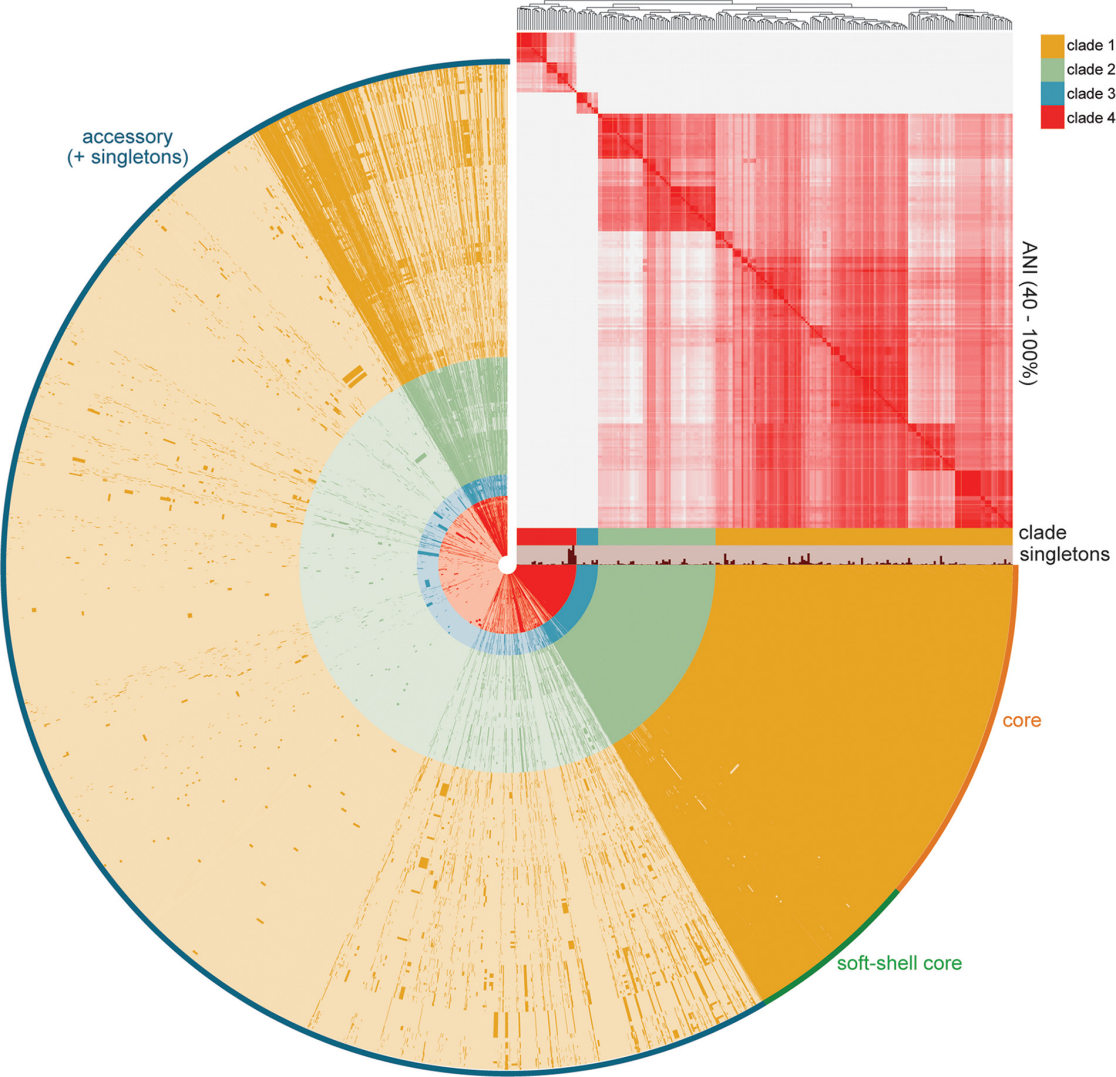
C. innocuum exhibits an open pangenome. C. innocuum pangenome displaying presence or absence of core (orange; present in 100% of genomes), soft-shell core (green; present in 95 to 99% of genomes), and accessory (blue; present in <95% genomes) genes, with hierarchical clustering based on average nucleotide identity (ANI) in the right-hand corner (coloring based on 40 to 100% similarity). Clade and cluster designated in the legend. Analyses incorporate all available unique C. innocuum genomes (*n* = 232).

The average nucleotide identity (ANI) (52) computed across all genomes revealed four distinct clades. Clades III and IV were 90% or less similar to clades I and II, less than an expected cutoff for a genus (90%)- or species-level (95%) comparison (53, 54) (Fig. 2). Clades I and II were more closely related with >95% ANI, and they included all four available reference genomes. We then used Anvi’o to assign core (present in all), soft-shell (present in 95 to 99% of genomes), and accessory genes (present in less than 95% of the genomes), which totaled 524 core and 28,915 accessory genes across all 232 genomes (Fig. 2). When excluding clade III and IV genomes, these totaled 2,058 core and 18,256 accessory genes, with the pangenome still open as indicated by the alpha and gamma parameters (Fig. S3).

10.1128/msphere.00569-22.5 FIG S3 The pangenome for canonical C. innocuum strains is open. (A) Total number of genes as a function of number of genomes within clades I and II (*n* = 193). Yellow-colored boxplots represent the average number of total genes from five subsamplings. The function follows Heap’s law (top left corner). The alpha is from Heap’s law estimate, run over 500 iterations using micropan in R (*P* < 2e-16). (B) Total number of unique genes as a function of number of genomes (*P* < 2e-16). (C) Core, soft-shell core, and accessory/singleton gene representation of the pangenome in clade I (green) or clade II (yellow) C. innocuum strains. Download FIG S3, TIF file, 2.7 MB. Copyright © 2023 Bhattacharjee et al. 2023 Bhattacharjee et al. https://creativecommons.org/licenses/by/4.0/ This content is distributed under the terms of the Creative Commons Attribution 4.0 International license.

**FIG 3:**
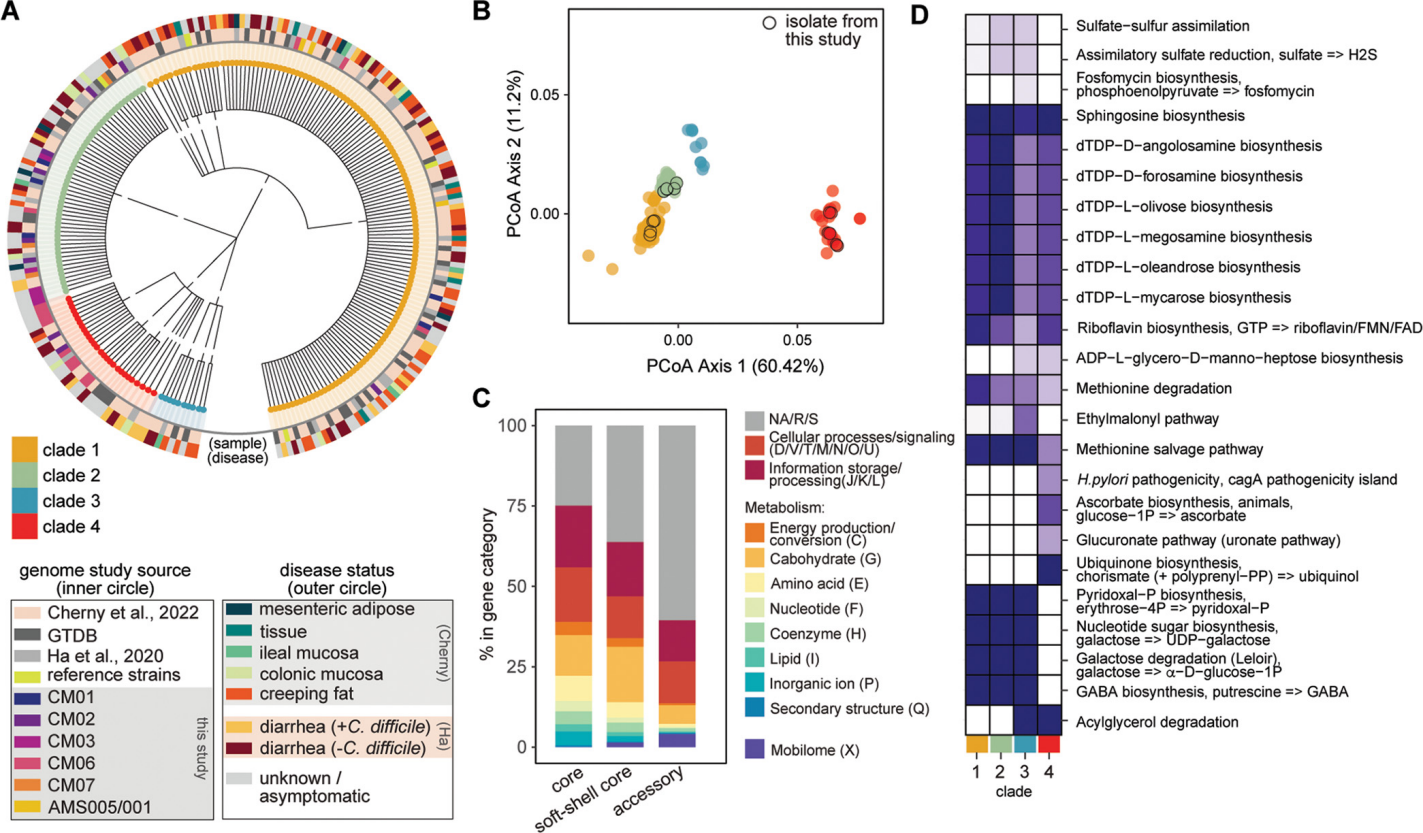
Clade-specific differences in metabolism and potential virulence drive genomic differences in C. innocuum strains. (A) Maximum-likelihood tree based on single nucleotide polymorphisms in 524 core genes from 500 replications, colored by clade (node color) and source (circle color) as specified in the legend. (B) Principal-coordinate analysis (PCoA) based on a Bray-Curtis distance matrix of COG gene assignments (presence or absence) generated using Prokka, colored by clade (legend) (PERMANOVA; **, *P* < 0.001). (C) Relative abundance of major COG categories (color coded in the legend) represented in core, soft-shell core, and accessory genes. (D) Differentially enriched KEGG modules across clades (false-detection rate using an adjusted *q* value below 0.05), colored by fraction of genomes within each clade.

A maximum-likelihood tree using the core genome of all strains reiterated clustering of C. innocuum genomes into four clades as observed by ANI (Fig. 3A), demonstrating widespread distribution of the isolates from this study across clades I, II, and IV. Additionally, there was not an observable overrepresentation of C. innocuum strains isolated from clinical cases within any clade. A maximum-likelihood tree based on a set of 400 selected protein markers present across all bacteria and archaebacteria constructed using PhyloPhlAn (55, 56) maintained the overall integrity of the four clades, with some shuffling between the closely related clades I and II (Fig. S1B).

### Genomic differences within C. innocuum and related strains are driven by metabolism.

Principal-coordinate analysis (PCoA) based on Bray-Curtis dissimilarity of presence or absence of COG genes demonstrated significant clustering of strains by clade (*P* < 0.001, permutational multivariate analysis of variance [PERMANOVA]), with clades I, II, and III closer together than clade IV (Fig. 3B). Overall, the pangenome of C. innocuum displayed ~28,000 gene clusters categorized using the COG database (Fig. 3C). At least 25% of the core genome and 50% of the accessory genes were classified as general functions (R), unknown functions (S), or did not map to the COG database (NA). Genes responsible for basic cellular processes and information storage and processing were equally distributed between the core, soft-shell core, and accessory genomes. Genes involved in metabolism (C, G, E, F, H, I, and P) were highest in the core genome and included genes associated with basic energy-producing pathways like ATP synthesis, gluconeogenesis, tricarboxylic acid (TCA) cycle, urea cycle, or Entner-Duodonoff pathway. From these functions, carbohydrate metabolism (G) held the highest percentage across all gene categories and was increased in the soft-shell core genes.

Interestingly, genes in the mobilome COG category (X) were overrepresented in the accessory genome of C. innocuum. Core mobilome representation included only a single mobilome gene, bacteriophage protein gp37, which forms a fibrous parallel homotrimer at the end of the long tail fibers in bacteriophages (57). Some phage-related genes, such as phage-related holin belonging to COG4824, which also harbors C. difficile TcdE (lysis protein), killer protein of prophage maintenance systems (Doc), predicted transposases (InsQ, InsG, and Tra8), and competence proteins (ComCG), were present in the soft-shell genome. The majority of the mobilome genes were present in the accessory genome, comprising a multitude of predicted transposases, transcription/translation proteins, and phage-related regulatory proteins. This included a plasmid stabilization system protein, ParE, identified across clades I, II, and IV strains, which, in *Enterobacteriaceae*, confers heat and antibiotic tolerance by maintaining IncI- and IncF-type plasmids and a DNA damage-inducible protein D, which has a role in recombinational DNA damage repair, as seen in Escherichia coli (58, 59).

To identify completeness of metabolic pathways (>75% of total genes), we used Anvi’o for metabolic reconstruction of the strains using the KEGG database. Completed carbohydrate metabolism pathways across all genomes (Fig. S4A) included the pentose phosphate pathway, pyruvate oxidation, glycolysis, glycogen biosynthesis and degradation, Embden-Meyerhof pathway, and ascorbate degradation pathway. All four clades demonstrated incomplete TCA cycle pathways. Several completed amino acid metabolism pathways were identified across all clades (including valine, proline, threonine, tryptophan, leucine, isoleucine, arginine, and ornithine) except for clade III, which also demonstrated a complete module for lysine (Fig. S4B). For lipid metabolism, completed pathways included fatty acid biosynthesis, initiation, and elongation (Fig. S4C).

10.1128/msphere.00569-22.6 FIG S4 Complete modules present across C. innocuum clades. Completion of modules associated with carbohydrate (A), amino acid (B), and lipid metabolism (C). Data were obtained by estimating metabolism using Anvi’o (87). Yellow refers to an incomplete module (<75% of the genes required to complete that pathway are present across all strains in their respective clades), and blue refers to a complete module (>75% of the genes required to complete that pathway). Download FIG S4, EPS file, 1.0 MB. Copyright © 2023 Bhattacharjee et al. 2023 Bhattacharjee et al. https://creativecommons.org/licenses/by/4.0/ This content is distributed under the terms of the Creative Commons Attribution 4.0 International license.

We used the Anvi’o functional enrichment tool to identify differentially abundant KEGG modules across clades (*P* < 0.05). Overall, clade I and II genomes exhibited similar profiles to each other compared to clade III and IV, although differences were observed between clades III and IV genomes (Fig. 3D). Clades I and II shared genes related to biosynthesis of terpenoids and polyketides that were less represented in clades III and IV. The glucuronate (uronate), ascorbate biosynthesis, and ubiquinone biosynthesis pathways were almost exclusively represented within clade IV strains compared to other clades. These pathways consisted of only single KOfam assignments belonging to the pathways UDP-glucose-6-dehydrogenase (EC 1.1.1.22), flavin prenyltransferase (EC 2.5.1.129), and xylulokinase (EC 2.7.1.17).

While module representation across all strains suggests that lipid biosynthesis and utilization are prevalent within C. innocuum (Fig. S3), acylglycerol degradation (triacylglycerol lipase [EC 3.1.1.3]) was observed for strains only in clade III and IV. Our analysis included C. innocuum genomes (SRA accession nos. SRR12535151 and SRR12535143) recently isolated from creeping fat in patients with Crohn’s disease as belonging to clade IV (40). A Helicobacter pylori *cagA* pathogenicity island signature module (K02283) was also identified as differentially represented in clade IV genomes. This includes the type IV pilus assembly protein CpaF (EC 7.4.2.8).

**FIG 4:**
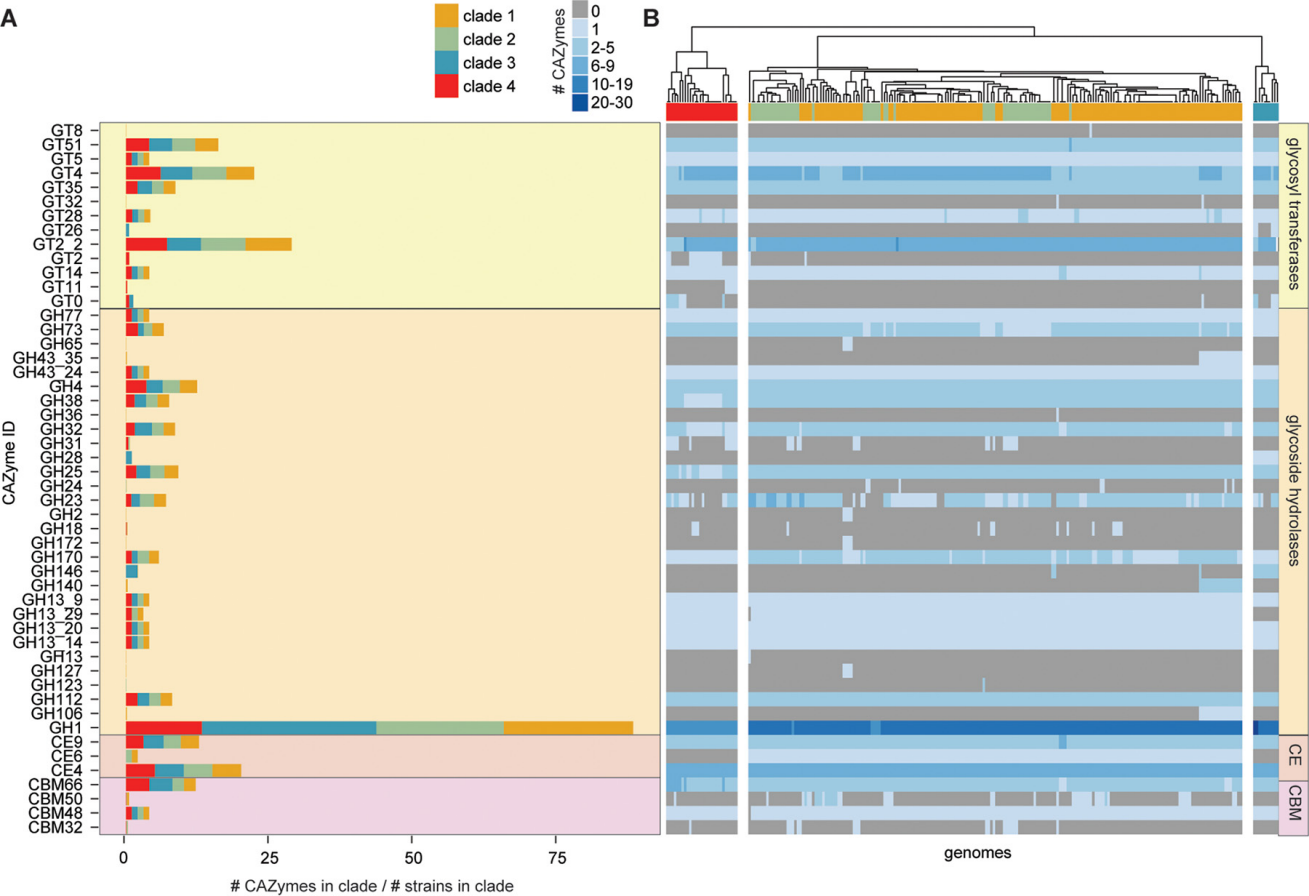
C. innocuum strains display clade-specific carbohydrate-activating enzymes (CAZymes). (A) Number of CAZyme types detected normalized to the total number of genomes within each clade, color coded by clade type. (B) Heatmap of number and type of CAZymes in individual genomes, clustered using Euclidean distance. GT, glycosyltransferase; GH, glycoside hydrolase; CE, carbohydrate esterase; CBM, carbohydrate-binding module.

To identify more specialized polysaccharide metabolism in C. innocuum, we used dbCAN and the CAZy database to identify carbohydrate-active enzymes (CAZymes) (60). Glycoside hydrolases in the GH1 group and GT2.2 glycosyl transferases were the most abundant CAZymes in C. innocuum collectively, albeit with some variability across the four clades (Fig. 4A). GT14, which produces a glycogen-branching protein, was present in all clades, while GT11, a fucosyltransferase, was only present in clade IV. Compared to clade II strains, strains in clade I were more likely to contain glycoside hydrolases involved in alpha- or beta-d-glycosidic bond hydrolysis (GH2, GH43_35, GH65, GH106, GH140, and GH36). GH146, a β-l-arabinofuranosidase that cleaves β-l-Araf bonds in plant pectins, was present in all clade III strains and a subset of clade I (61). The acetyl-mannosamine transferase GT26 was only present in clade III strains. Only three types of carbohydrate esterases (CEs) were identified across all C. innocuum strains, all involved in plant cell degradation, CE6 (acetyl xylan esterase), CE4 (chitin deacetylase), and CE9 (*N*-acetylglucosamine 6-phosphate deacetylase) (62, 63). Four carbohydrate-binding modules were also observed across all clades, including CBM66, part of the LPXTG cell wall anchor domain-containing protein that degrades fructoside residues in fructans, and CBM48, associated with the GH13 family of CAZymes responsible for degrading starches (64). Other carbohydrate-binding modules, such as CBM50, which is often associated with GH25 to degrade chitin or peptidoglycan (65), and CBM32, involved in galactose and/or lactose metabolism, were observed sporadically across clades I, II, and IV.

### C. innocuum exhibits strain-level variation in substrate use *in vitro*.

To examine differences in nutrient use across strains, we selected seven representative strains to examine their ability to use distinct carbohydrate sources *in vitro*. Representative strains were selected from a 99% dereplication cutoff of C. innocuum genomes, which clustered the genomes into seven groups, representing three of the four clades. We assessed both growth and acid production of the strains in minimal media supplemented with single carbohydrates. After 24 h of growth assessment, acid production was assessed using the colorimetric pH indicator bromocresol purple (BCP), which approximates pH changes as a result of fermentation (Fig. S5). Acid production signifying fermentation was determined as low (pH 5.5 to 6.5 and optical density at 588 nm [OD_588_] of 0.26 to 0.44) or high (pH < 5.5 and OD_588_ < 0.26).

10.1128/msphere.00569-22.7 FIG S5 Absorbance values and pH exhibit a strong correlation in BMCA for bromocresol purple (BCP) assay. (A) Correlation between absorbance values (OD_588_) of BCP and pH. Dark blue represents the linear regression of absorbance versus pH for rich media (TCCFB) control. Light blue represents the linear regression of absorbance versus pH for basal media (BMCA). An *R*^2^ of 0.94 indicates a strong correlation between absorbance (OD_588_) of BCP to pH in BMCA. (B) Regression equation for BMCA used to predict pH values for strains grown in BMCA containing one carbohydrate source as a predictor of bacterial growth. (C) Actual pH values used to confirm expected values calculated from BMCA linear regression. Download FIG S5, EPS file, 1.3 MB. Copyright © 2023 Bhattacharjee et al. 2023 Bhattacharjee et al. https://creativecommons.org/licenses/by/4.0/ This content is distributed under the terms of the Creative Commons Attribution 4.0 International license.

**FIG 5:**
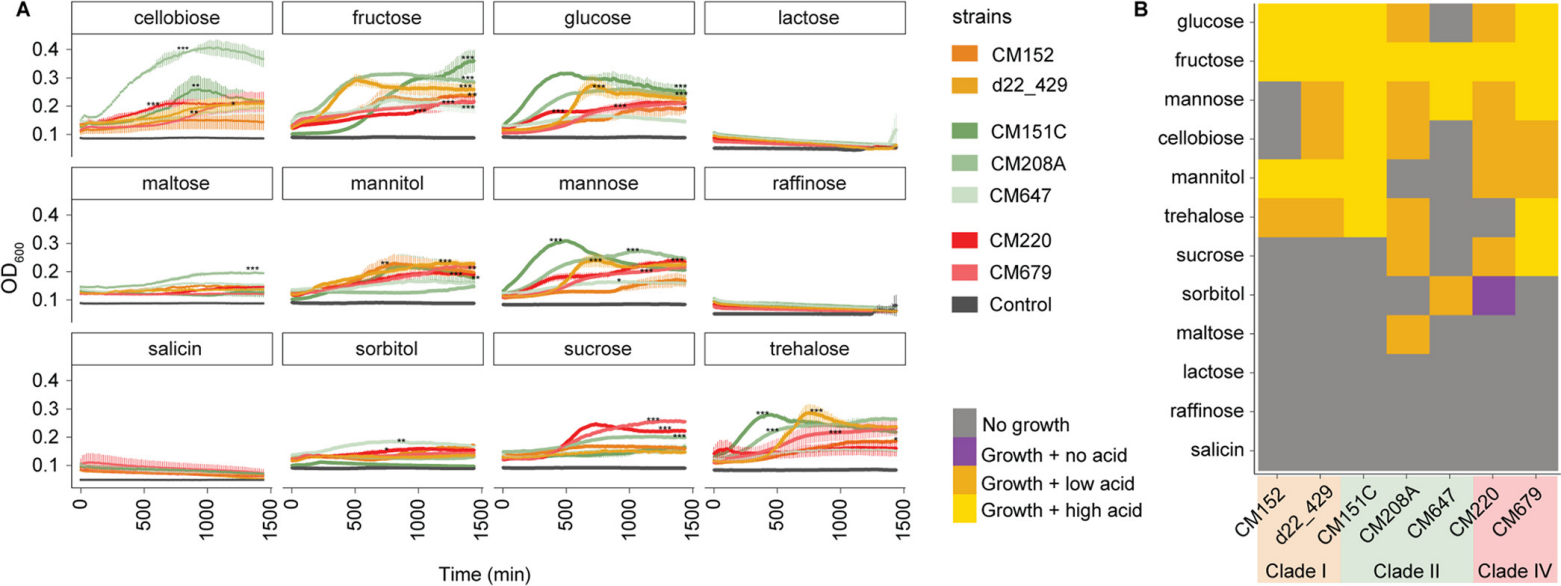
C. innocuum exhibits strain-specific differences in nutrient utilization *in vitro*. (A) Growth curves of strains (*n* = 7) inoculated into basal medium with Casamino Acids (BMCA) with indicated carbohydrate source over 24 h, measured at OD_600_. Gray indicates the negative control (strain inoculated into BMCA without addition of carbohydrate). The significance of growth was determined by ANOVA on area under curve per strain (within each sugar type), followed by Tukey’s honestly significant difference (HSD); *, *P* < 0.05; **, *P* < 0.005; ***, *P* < 0.0001. (B) Growth (from OD_600_) and acid production (from bromocresol purple assay) data were combined to show nutrient utilization patterns in representative strains. High acid production was classified as pH of <5.5 and OD of <0.26; low acid production was classified as pH 5.5 to 6.5 and OD_588_ of 0.26 to 0.44 (legend). Clade and strain designation indicated by the legend.

We observed variation in the ability of strains to grow on minimal media supplemented with single carbohydrate sources (Fig. 5). None of the strains could grow on lactose or raffinose, confirming previous observations for C. innocuum (27, 42). In contrast to previous reports (32), salicin did not support growth of any of the strains tested. Most strains demonstrated high growth (defined by both significant increases in OD and high acid production) in glucose and fructose, albeit at various growth rates. Significant growth on glucose and mannose was observed by all but one strain in each, CM647 and CM152, respectively (analysis of variance [ANOVA] on the area under the concentration-time curve [AUC], *P* < 0.05). Cellobiose, mannitol, and trehalose all supported growth and acid production in most strains (ANOVA, *P* < 0.05). Maltose supported the growth of only one strain, CM208A (ANOVA, *P* < 0.05), whereas sorbitol only supported growth of two strains, CM647 and CM220 (ANOVA, *P* < 0.05).

Only some of the variable growth aligned with their clade designation. Within clade I, both CM152 and d22_429 followed a similar pattern of acid production (Fig. 5B) and grew at various efficiencies in glucose, fructose, mannitol, and trehalose (Fig. 5A). Within clade IV, both strains CM220 and CM679 grew efficiently in several carbohydrates and more efficiently on sucrose than most strains (ANOVA, *P* < 0.0001). The most variation was observed in clade II strains, with CM647 consistently displaying minimal growth on most carbohydrates. In contrast, both CM208A and CM151C exhibited some of the highest growth in glucose, cellobiose, and mannose compared to other strains (ANOVA, *P* < 0.005), but CM208A did not grow in mannitol compared to CM151C (ANOVA, *P* < 0.005). CM208A also consistently produced less acid than CM151C, except for growth in fructose (Fig. 5B).

### C. innocuum exhibits clade-level variation in putative virulence factors and toxins.

**FIG 6:**
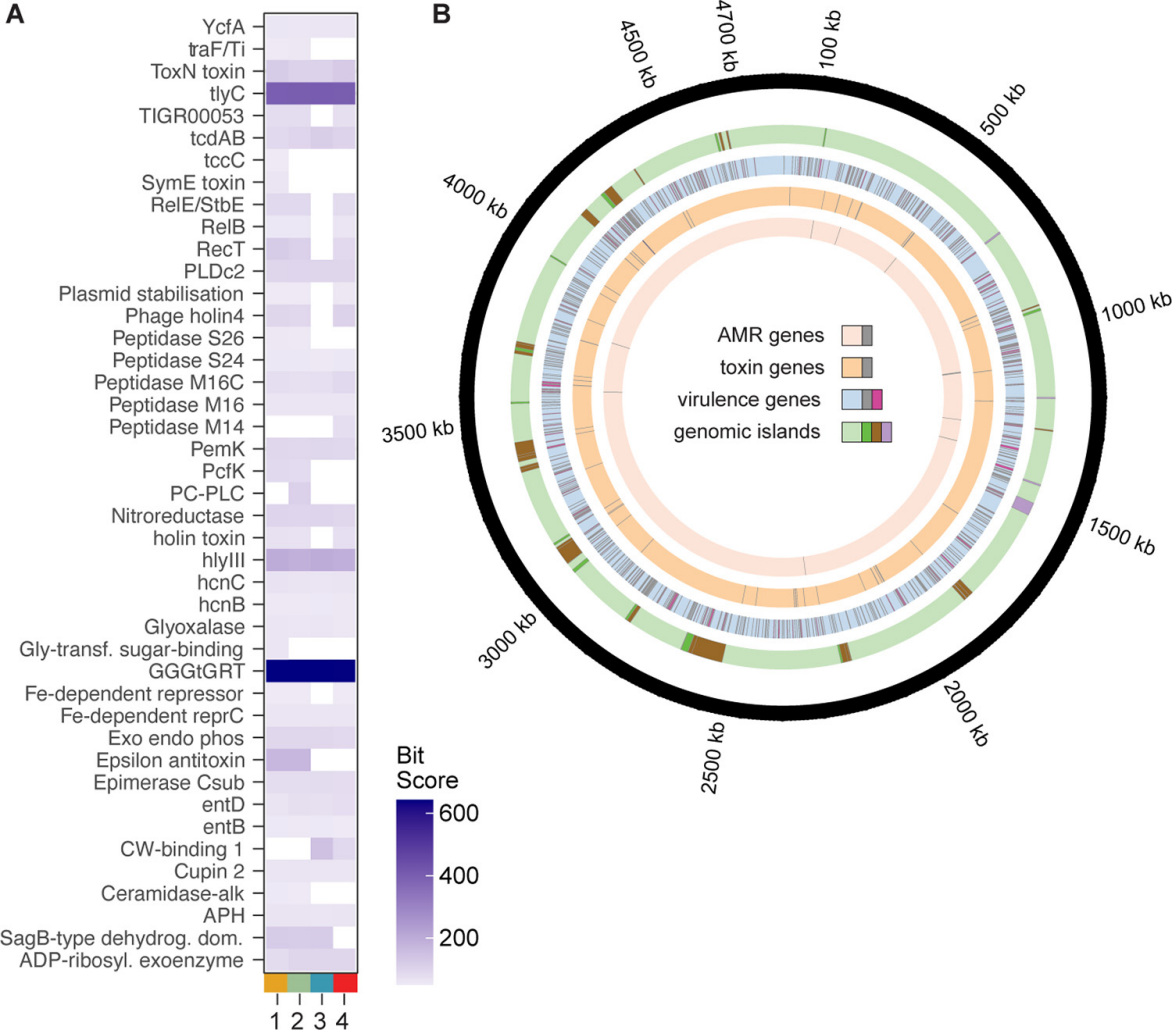
Certain clades of C. innocuum display enhanced potential virulence. (A) Average bit score of select putative secreted and nonsecreted toxins (bit score > 50) across C. innocuum clade, identified using PathoFact. (B) Location of antibiotic resistance genes (AMR), putative virulence factors, and toxins identified using PathoFact (three innermost circles) and genomic islands, identified using IslandViewer 4 (penultimate outer circle; green, forward orientation; brown, reverse; purple, bidirectional orientation).

An exotoxin has not been identified from C. innocuum despite previous evidence of association with infection (66). Using PathoFact to identify potential virulence (bit score > 50), we observed differential distribution of putative virulence factors across the four clades (Fig. 6A) (67). Overall, clade III strains had lower numbers of virulence factors detected over the other clades, which also lacked the type II toxin-antitoxin system from the YafQ/RelB/ParE family and phage lysis holins. Some factors were present across all clades, including members of hemolysin III, *hlyIII* and *tlyC*; NlpC/P60 family, *pspA* and *pspC* (identified as *entD* in the software); and a type III toxin-antitoxin system from ToxN/AbiQ (Fig. 6A). GGGtGRt, *tlyC*, and *hlyIII* demonstrated the highest bit scores across all clades, including within the reference strain C. innocuum 14501. While we identified the presence of C. difficile *tcdAB*-like genes in all clades, a BLAST search across the NCBI databases revealed that they likely belong to the NlpC/P60 family of proteins, either as surface protein *pspAC* alongside a penicillin-binding protein *mrcB* or as a glucan-binding domain-containing protein, not yet fully characterized. A PCoA based on the Bray-Curtis dissimilarity distance from the presence or absence of putative virulence genes from C. innocuum strains isolated from either diarrheal patients or healthy controls (*n* = 119) (46) did not demonstrate clustering based on clinical status (Fig. S7), suggesting that no groups or single putative toxins were associated with disease. Additionally, using functional enrichment for general pathways (Anvi’o) within this genome set did not identify significantly enriched KEGG or COG classes.

10.1128/msphere.00569-22.8 FIG S6 Predicted presence or absence of antimicrobial resistance genes in C. innocuum. Antimicrobial resistance gene categories identified by PathoFact in C. innocuum clades (black, presence of a gene in the antibiotic resistance gene category in at least one strain). Download FIG S6, EPS file, 0.5 MB. Copyright © 2023 Bhattacharjee et al. 2023 Bhattacharjee et al. https://creativecommons.org/licenses/by/4.0/ This content is distributed under the terms of the Creative Commons Attribution 4.0 International license.

10.1128/msphere.00569-22.9 FIG S7 C. innocuum strains isolated from Clostridioides difficile diarrhea patients do not cluster by gene content. Principal-coordinate analysis (PCoA), based on a Bray-Curtis distance matrix, made from the presence or absence of COG gene assignments generated using Prokka shaped by clade and colored by disease association (legend) within C. innocuum strains isolated from C. difficile-diarrheal patients in Cherny et al. (46). PERMANOVA, performed using adonis2 (vegan) in R, indicated significant correlation with clade assignment (**, *P* < 0.001) but no significant correlation with disease. Download FIG S7, TIF file, 2.8 MB. Copyright © 2023 Bhattacharjee et al. 2023 Bhattacharjee et al. https://creativecommons.org/licenses/by/4.0/ This content is distributed under the terms of the Creative Commons Attribution 4.0 International license.

The chromosomal location of virulence and antibiotic resistance genes identified by PathoFact were visualized in conjunction with genomic islands (using IslandViewer 4), with C. innocuum 14501 as a reference (Fig. 6B) (68). Resistance genes against vancomycin glycopeptide (*vanRS*), tetracycline, as well as several ABC transporters and aminoglycoside genes were identified in all clades. Bacitracin resistance (*bcrAC*) was found only in clades I and III (Fig. S6). Results from IslandViewer 4 predicted 42 genomic islands, with 113 virulence factors and 7 toxin genes aligning with genomic island locations (Fig. 6B). While none of the hemolysins aligned with genomic island predictions, a type II TA system involving RelE/StbE family of toxin-antitoxin system (with 11 additional genes) and a group of NlpC/P60 glucan-binding proteins (labeled as *tcdAB* by PathoFact, with 9 additional genes), each aligned with a predicted genomic island.

## DISCUSSION

To date, this study marks the most comprehensive characterization of genomic variability within the human gut inhabitant C. innocuum. While C. innocuum has originally been designated a commensal from initial isolation studies (32, 34), it has also been recently associated with various disease states (29, 37–40). We recovered C. innocuum strains from all individuals sampled in this study, suggesting a high prevalence of C. innocuum in the “healthy” human gut. This is further strengthened by the prevalence of C. innocuum in human 16S rRNA gene-based surveys using a custom classifier specific for C. innocuum 16S rRNA sequences, which also identified increased abundance of C. innocuum following antibiotic use. These data, in addition to the consistent association of C. innocuum with disease in culture-based studies, support a closer look at the role of C. innocuum in the gut microbiota.

Our genomic analysis of C. innocuum clarifies some of the functions attributable to C. innocuum colonization of the gut. We identified several complete modules in both carbohydrate and amino acid metabolism within the C. innocuum core genome. These also included multiple genes associated with utilization of saccharides, such as glucose, mannose, fructose, xylose, mannitol, chitin, xylan, and other starches and peptidoglycans, indicating an ability to use plant polysaccharides directly or by-products of polysaccharide degradation by other commensals. Additionally, all C. innocuum strains exhibited several partial and complete modules for lipid metabolism. None of the tested strains were able to grow in lactose, salicin, and raffinose, corroborating descriptions of C. innocuum clinical isolates growth using a Biolog platform (40, 69).

Our genomic comparison also revealed strain-specific diversity in C. innocuum. Both ANI and phylogenetic analysis of C. innocuum genomes demonstrated four distinct clusters. Clades III and IV were, collectively, 90% or less similar to clades I and II, which contained all available C. innocuum reference strains, suggesting that these clades may represent a new erysipelotrichial species related to C. innocuum. Even after exclusion of clade III and IV genomes, the pangenome of C. innocuum remains highly open as assessed by Heap’s law (70). It has been suggested that an open genome may reflect a more sympatric lifestyle, whereby related species interacting with each other can easily share genetic elements (71). While our current study did not specifically focus on identification of mobile elements, most mobilome-related genes were present in the accessory portion of the C. innocuum pangenome, suggesting a high degree of horizontal gene transfer among C. innocuum and the two related species.

The ability to acquire new genes can provide a competitive metabolic advantage in a microbially dense environment. As the nutrient niche theory stipulates, colonization by an invading microbe, pathogenic or commensal, is at least partially dependent on its ability to better utilize nutrients to outcompete extant microbes in that environment (72). For example, coexistence of the highly abundant gut inhabitant Bacteroides thetaiotaomicron is likely possible at least in part due to the diversity of polysaccharide-utilizing loci observed across different strains that allow flexibility in resource utilization (73). We observed coexistence of several C. innocuum strains within a single fecal sample, none of which were 100% identical to each other, and some of which spanned multiple clades within an individual. Overall, the CAZymes observed across C. innocuum were fewer than previously characterized gut commensals (13, 74, 75), with some clade-specific CAZymes. These genome-encoded CAZyme differences between clades and potential new species could indicate niche partitioning to support related strains within the same environment or the ability to localize into distinct gastrointestinal locations.

The realized, or expressed, niche of strain coexistence is likely more complicated (76). Our *in vitro* growth assays support niche partitioning within the canonical C. innocuum clades I and II at both clade- and strain-specific levels. For example, both CM152 (clade I) and CM151C (clade II), isolated from the same individual, could use fructose, glucose, mannose, and trehalose for growth. Yet the former grew significantly better with fructose, whereas the latter grew better on glucose, mannose, and trehalose. Despite the observation of clade-specific genomic patterns in CAZymes, we did not observe overt clade-specific growth patterns *in vitro*. This suggests that realized metabolic niche partitioning can occur within an individual beyond the categorical genomic features assessed, emphasizing the importance of regulatory or additional genes that contribute to successful coexistence of related strains. These differences also likely influence or are influenced by other members of the collective microbiota within an individual.

The demonstrated genomic and phenotypic variability observed across the C. innocuum species may also be of clinical importance. C. innocuum is commonly isolated in conjunction with gastrointestinal tissue or fecal clinical samples (35, 40, 66). A recent retrospective study in a Taiwanese clinical cohort isolated C. innocuum rather than C. difficile from patients with C. difficile-like clinical presentation (35). The C. innocuum isolates in this study were reported to cause a range of cytotoxicity and enteropathogenic effects *in vitro*. Most recently, Cherny et al. reported that C. innocuum isolates from pediatric patients enrolled in C. difficile studies cross-reacted with the enzyme immunoassay (EIA) diagnostic test for C. difficile toxins A and B (66). The study identified a putative C. innocuum toxin EIA cross-reactive factor (ErF) similar to the NlpC/P60 family of toxins in all isolates tested but observed no cytotoxicity. We identified the same putative toxin A/B gene in clades I and II, with a significantly higher similarity score for tcdA/B in clade II genomes. C. innocuum has also been postulated as a potential causative agent of “creeping fat,” an extraintestinal phenomenon correlated with Crohn’s disease (CD) (40). Ha et al. demonstrated that C. innocuum isolated from various human intestinal mucosal locations could translocate into tissue in a mouse model of inflammatory bowel disease (IBD). Our analysis, which included genomes from that study, did not identify clade-specific clustering based on the anatomical site. However, the two clade IV representatives identified as part of this study were both isolated from creeping fat lesions. Yet genomic content, either comprehensively or within a subset of putative virulence factors, did not correlate with disease status (46). Furthermore, genomes associated with disease state (either with C. difficile or in association with “creeping fat” in CD) spanned the four clades identified in this study, demonstrating no definitive “virulent” strain. Together, these data suggest the possibility of C. innocuum or a closely related species as an opportunistic, rather than an absolute, pathogen.

In summary, we demonstrate strain-specific variation of a prevalent gut “commensal” that, until recently, was considered relatively benign in the gut environment. The increased association of C. innocuum with gastrointestinal conditions supports further investigation of the role of C. innocuum in the gut, with an emphasis on identification of novel virulence or invasive factors that might enable C. innocuum to cause disease. Furthermore, our results reveal the importance of understanding strain variation that can be extended to other gut commensals.

## MATERIALS AND METHODS

### Isolation of C. innocuum.

This study was approved by Clemson University’s Institutional Review Board. Healthy donors were over 18, had not taken antibiotics or been diagnosed with any infections within 6 months, and were not immunocompromised or diagnosed with chronic gastrointestinal conditions. Upon receipt, fecal samples were placed under anaerobic conditions (Coy Laboratory Products, Grass Lake, MI, USA; 85% nitrogen, 10% hydrogen, and 5% carbon dioxide) and streaked onto brain heart infusion (BHI) (77), BHI supplemented with fetal bovine serum (FBS; 50 mL/L BHI), or taurocholate cycloserine-cefoxitin-fructose (TCCFA) (78, 79) by using different streaking strategies. Streaks were incubated at 37°C for at least 24 h and then picked and streaked for purity. Samples were stored at −80°C in 20% glycerol stocks for future *in vitro* work or DNA extraction (see Text S1 in the supplemental material).

10.1128/msphere.00569-22.1 TEXT S1 Supplemental materials and methods containing detailed methodology on medium preparation; C. innocuum isolation pipeline; bromocresol purple assay; and whole-genome assembly pipeline, phylogeny, pangenome analysis, CAZyme analysis, and virulence factor analysis. Download Text S1, DOCX file, 0.1 MB. Copyright © 2023 Bhattacharjee et al. 2023 Bhattacharjee et al. https://creativecommons.org/licenses/by/4.0/ This content is distributed under the terms of the Creative Commons Attribution 4.0 International license.

### DNA extraction and identification of C. innocuum.

All isolates were heat extracted at 95°C for 20 min for PCR using Go Taq (Promega; catalog no. M7132) and 8F and 1492R primers to amplify the whole 16S rRNA gene (4). PCR products were cleaned up using) and sent to Eton Biosciences for Sanger sequencing, using EzBioCloud and RDP databases Illustra ExoProStar kit (Cytiva; catalog no. US78210for identification (80, 81). DNA extraction for sequencing was performed from 1.8 mL of overnight culture using the Qiagen DNeasy UltraClean microbial kit (Qiagen; catalog no. 12224-250). Extracted DNA was diluted to 10 ng/μL concentration (Qubit, Life Technologies; catalog no. Q33230) and sent to the Microbial Genome Sequencing Center (MiGS), Pittsburgh, Pennsylvania (https://www.seqcenter.com/), for Illumina sequencing using the NextSeq 2000 platform.

### Prevalence of C. innocuum in human 16S rRNA gene-based surveys.

FASTA sequences of full-length 16S rRNA sequences from C. innocuum genomes were formatted for alignment in mothur (43) and aligned using the SILVA database (v132) (82). Previously published sequences from fecal microbiota samples representing healthy (83), hospitalized, and/or septic (84) or antibiotic-exposed (85) individuals were processed in mothur using the Schloss lab standard operating procedure (SOP), aligning to the SILVA database (44) and then classifying to the custom classifier using the classify.seqs command in mothur (cutoff = 95) or directly to the RDP database (v16) for comparison (86). The log_10_ relative abundance of C. innocuum was plotted in R using the Kruskal-Wallis test in R with a pairwise Wilcoxon rank-sum test for pairwise comparisons between groups.

### *In vitro* growth of C. innocuum.

Representative strains were chosen from all available C. innocuum strains based on 99% dereplication using pyANI in anvi-dereplicate-genomes (52, 87). Strains from freezer stocks were initially streaked onto TCCFA in an anaerobic chamber and incubated at 37°C for 24 h. A single colony was added into 4 mL TCCF broth (TCCFB) and incubated at 37°C for 24 h. We centrifuged 1.8 mL of overnight culture at 6,000 rpm for 5 min. Ten microliters of the resuspended pellet in 1.8 mL of prereduced water was added into wells of a 96-well plate (CoStar) containing 100 μL basal medium (88) with Casamino Acids (BMCA) with or without selected carbohydrate sources at 4% (wt/vol). A positive control of the resuspended strain in TCCF broth and BMCA without strain was included on each plate. The prepared plate was placed into a plate reader (Tecan Sunrise) for growth at 37°C, measuring the optical density at 600 nm (OD_600_) every 15 min for 24 h. A bromocresol purple (BCP) assay was used on the plate growth to assess pH (Text S1).

### Whole-genome assembly and phylogeny.

Full commands are available at https://github.com/SeekatzLab/C.innocuum-diversity and are further described in Text S1. Briefly, raw reads were quality checked and adapter trimmed using Trim Galore! (89) and assembled using SPAdes (90) as optimized with MEGAHIT (91). Quast with MultiQC was used to calculate assembly statistics (Table S1) (92, 93). Average coverage was calculated using Bowtie 2 and SAMtools (94, 95). Prokka was used to annotate assemblies (50). To verify the assembly identity, annotations were run through NCBI BLAST and EzBioCloud. Assemblies were also mapped onto the Genome Taxonomy Database (GTDB) (45) through GTDB-tk using classify_wf (96). Maximum-likelihood trees from the C. innocuum core genome SNP sites were determined by Roary using RaXML 8.2.12 with bootstrapping 500 times. The 16S rRNA maximum-likelihood tree was aligned using Clustal Omega and bootstrapped 500 times by RAxML (97, 98). The amino acid fasta phylogenetic tree was mapped against the PhyloPhlAn database with DIAMOND using PhyloPhlAn (55). Trees were visualized using GraPhlAn (99) or the ITOL web server (https://itol.embl.de/) (100–102).

### Pangenome analysis, functional enrichment, average nucleotide identity, and dereplication.

Contigs from SPAdes were reformatted and annotated with the COG and KEGG databases using Anvi’o v7.0 (87). Anvi’o was also used to create and visualize the pangenome, determine average nucleotide identity (ANI), and dereplicate strains within the data set (Text S1). Heap’s law was calculated in R (formulated as *n* = κ*N*^γ^, where *n* is the pangenome size, *N* is the number of genomes used, and κ and γ are the fitting parameters), and the α parameter was calculated using micropan (70, 103). ANI was computed using the anvi-compute-genome-similarity with pyANI (52). Dereplication between strains was computed using anvi-dereplicate-genomes at 90, 95, 98, 99, 99.9, and 100% similarity threshold. The false-detection rate correction for *P* values for functional enrichment was applied using the package qvalue from Bioconductor (104) and visualized using dplyr, ggplot2, and readxl packages (105–107). Principal-coordinate analysis and associated PERMANOVA statistics were performed in R using vegan and ape packages (108, 109).

### CAZyme and putative virulence.

CAZymes were predicted using dbCAN v2.0.6 (60), using the FASTA nucleotide sequences generated from Prokka for each of the strains. Prokka-generated nucleotide FASTA files (FNA) were processed through PathoFact (67) to predict virulence factors, toxins, and antimicrobial peptides. Genomic islands were predicted in C. innocuum 14501 using the web computational tool IslandViewer 4 (68). Circos was used to visualize chromosomal locations on reference sequence ATCC 14501 (68).

### Data availability.

All raw sequence data and associated information have been deposited in the NCBI Sequence Read Archive under BioProject accession no. PRJNA841489. All data analysis from raw sequence processing and additional data tables (containing information on dereplication reports, medium composition for *in vitro* studies, average number of new genes versus number of genomes, full list of bioinformatics resources, and list of antibiotic resistance genes) for the final manuscript are available at https://github.com/SeekatzLab/C.innocuum-diversity.
