# Supplementary figures and images for: Diversity and Prevalence of Clostridium innocuum in the Human Gut Microbiota
Source: mSphere. 2022 Dec 21;8(1):e00569-22. doi: 10.1128/msphere.00569-22 (PMC9942572; doi:10.1128/msphere.00569-22)

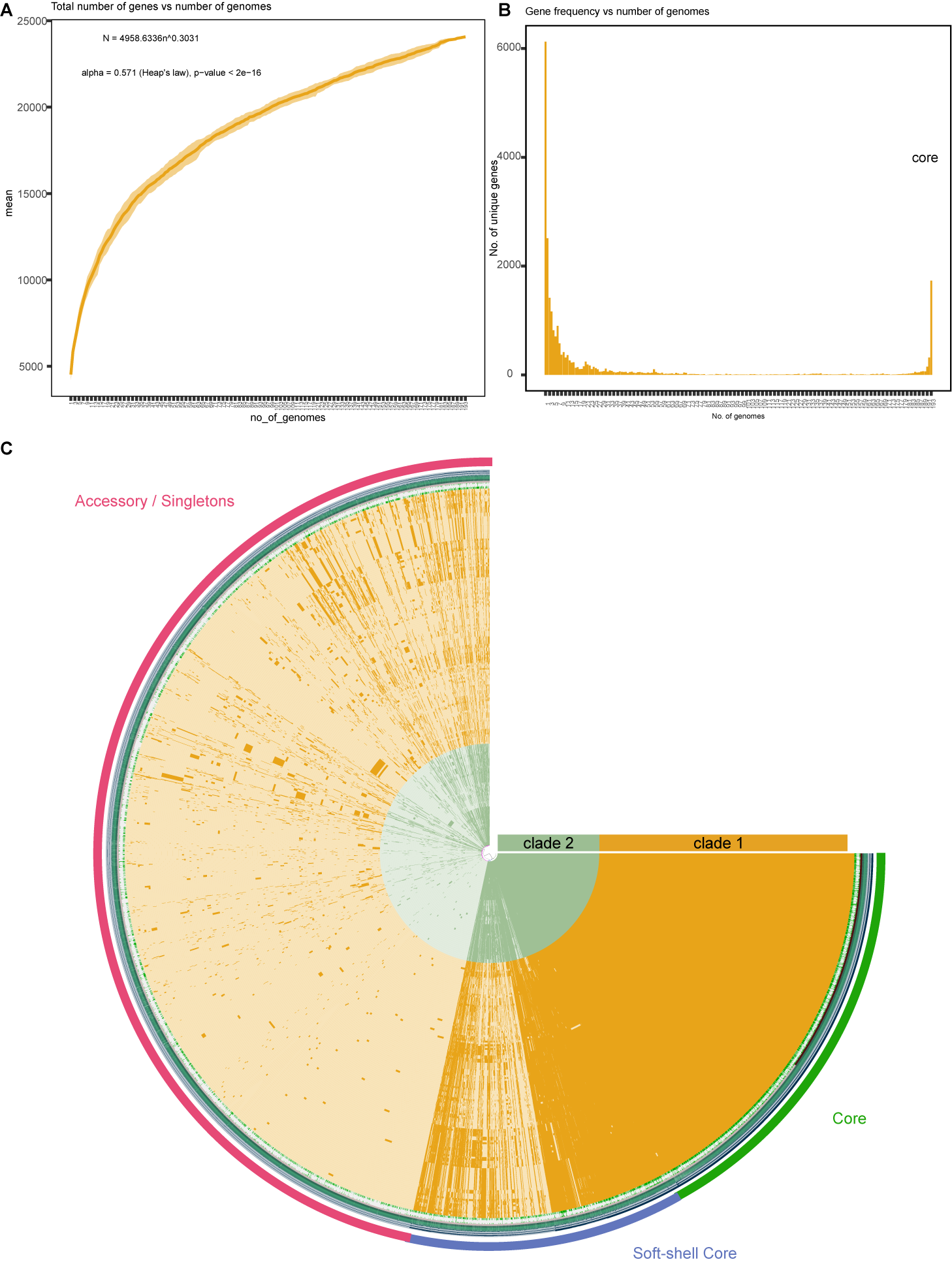

Supplement: FIG S3 [file msphere.00569-22-s0005.tif]

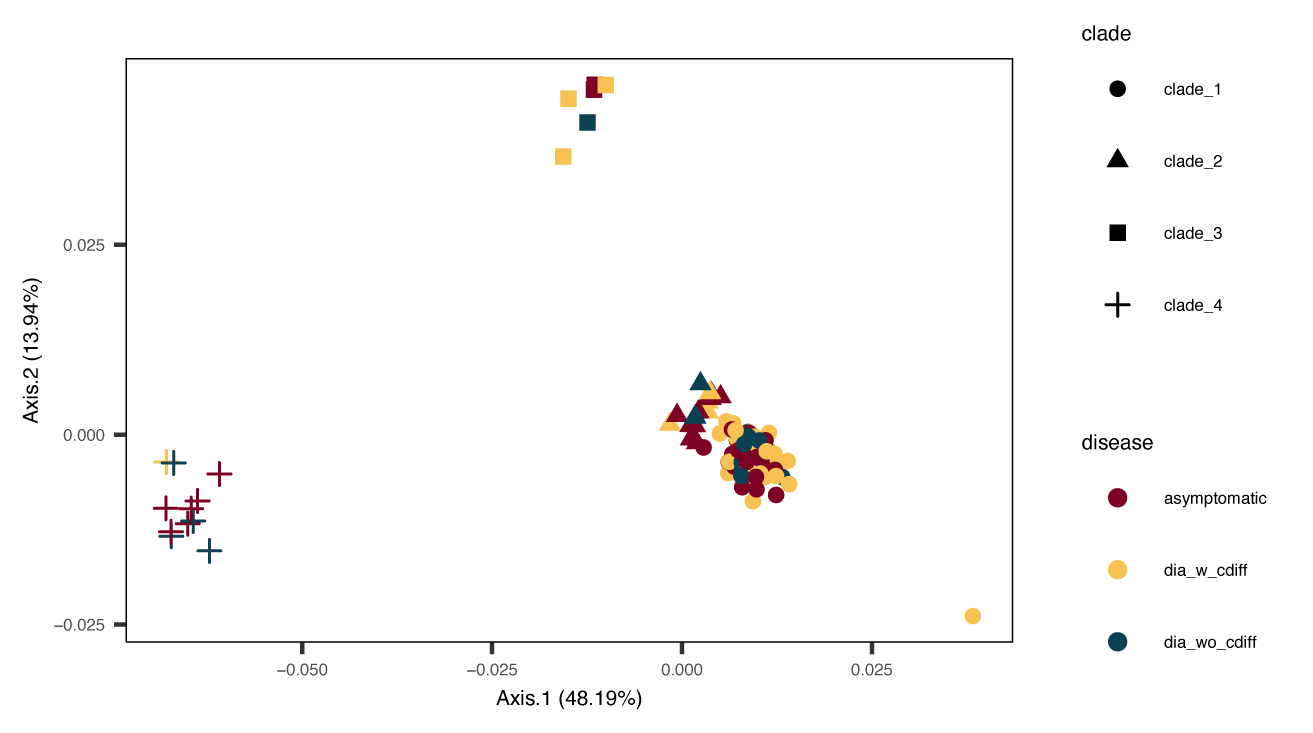

Supplement: FIG S7 [file msphere.00569-22-s0009.tif]
