# Supplementary material for: Diversity and Prevalence of Clostridium innocuum in the Human Gut Microbiota
Source: mSphere. 2022 Dec 21;8(1):e00569-22. doi: 10.1128/msphere.00569-22 (PMC9942572; doi:10.1128/msphere.00569-22)
Supplement: TEXT S1 [file msphere.00569-22-s0001.docx]

**Supplementary Materials and methods:**

**Media Preparation**

**To prepare 1 L of Taurocholate Cycloserine-Cefoxitin-Fructose (TCCFA) or TCCF broth:**

**Materials:**

Proteose Peptone No. 3 (BD Diagnostic Systems: 211693; VWR 90000-278)

Na_2_HPO_4_ (Fisher Scientific: 3828-01 or Sigma S5136)

KH_2_PO_4_ (Fisher Scientific: P285)

NaCl (Sodium chloride) (Fisher Scientific: S271-1)

MgSO4 (Sigma M7506)

D-Fructose (Fisher Scientific: L95-500)

Select agar (Invitrogen: 30391-023; Fisher 30-391-023)

D-cycloserine (Fisher Scientific NC0349872: Sigma C6880-5G)

Cefoxitin sodium salt (Sigma-Aldrich: C4786-1G)

Taurocholic acid sodium salt hydrate (Sigma-Aldrich: T4009)

Petri dishes (Fisherbrand: FB0875712)

25mm syringe filter (Fisherbrand: 09-720-004)

13mm syringe filter (Fisherbrand: 09-720-3)

30ml syringe (BD 302833)

10ml syringe (BD 309604)

Obtain 2L glass Erlenmeyer flask and rinse flask with Milli-Q water. Fill graduated cylinder with 800mL of Milli-Q water and add to the Erlenmeyer flask. Add stir bar to flask and place on stir plate, add:

40g Proteose Peptone No.3

5g Na_2_HPO_4_ (sodium phosphate dibasic)

1g KH_2_PO_4_ (potassium phosphate monobasic)

2g NaCl

0.1g MgSO_4_ - anhydrous

6g Fructose

20g Select agar (**do not add if making TCCFB)

Bring media up to 1000mL total in graduated cylinder and pour back in to 2L flask then add 1 mL resazurin solution (foil wrapped at 4°C). Cover flask with foil and autoclave tape and autoclave for 30min at slow exhaust-liquid. Once autoclaved, allow flask to cool down until cool to the touch and add:

- 1. 10mL of 10% (w/v) taurocholate (1g in 10mL water, filter sterilized, use 30ml syringe and 25mm syringe filter)
  2. 25mL D-cycloserine (10mg/mL, filter sterilized, use 30ml syringe and 25mm syringe filter) [0.25g into 25ml water]
  3. 1.6mL cefoxitin 10mg/mL, filter sterilized, use 13mm filter and 10ml syringe) [0.02g into 2ml water]

Once taurocholate, D-cycloserine, and cefoxitin have been added to the flask, media can be poured into petri dishes if making TCCFA plates or aliquoted into smaller sterile containers if preparing TCCF broth. After sufficiently cool media is ready to pour, clean work area with ethanol, allow to dry and light flame on Bunsen burner. Open media over flame and grasp flask with one hand and sterilize using the flame. Pour plates on even surface and avoid addition of bubbles, may get 30-50 plates total depending on how thick plates are poured. Make sure to fill bottom of petri dish. Once poured, replace top of lid on dish. Allow plates to dry on bench. Once the plates are dry, place them in the plastic sleeves and store at 4°C. Allow 24-48 hours for media to fully reduce if using for anaerobic work.

**To prepare 1L of Brain Heart Infusion agar or broth:**

**Materials:**

Brain Heart Infusion (BD 211059; VWR 90000-060)

Select agar (Invitrogen: 30391-023; Fisher 30-391-023)

Yeast Extract* (BD 212750; VWR 90000-726)

L-cysteine (Sigma C6852)

Petri dishes (Fisherbrand: FB0875712)

13mm syringe filter (Fisherbrand: 09-720-3)

10ml syringe (BD 309604)

Obtain 2L glass Erlenmeyer flask and rinse flask with Milli-Q water. Fill graduated cylinder with 800mL of Milli-Q water and add to the Erlenmeyer flask. Add stir bar to flask and place on stir plate, add:

37 g BHI

5 g Yeast agar* note: this is optional

15 g Select agar (** do not add this if making broth)

Bring media up to 1000mL with Milli-Q water in flask. Add 1 mL resazurin solution (foil wrapped at 4°C). Cover with foil and autoclave tape and autoclave for 30min at slow exhaust-liquid. Once autoclaved, allow flask to cool down until cool to the touch and add:

- 1. 10 mL of L-cysteine (0.1 g of L-cysteine in 10 mL water, filter sterilized, use 13 mm filter and 10mL syringe)

Once cysteine has been added to flask, media can be poured into petri dishes if making plates or aliquoted into smaller sterile containers if preparing broth. After sufficiently cool media is ready to pour, clean work area with ethanol, allow to dry and light flame on Bunsen burner. Open media over flame and grasp flask with one hand and sterilize using the flame. Pour plates on even surface and avoid addition of bubbles, may get 30-50 plates total depending on how thick plates are poured. Make sure to fill bottom of petri dish. Once poured, replace top of lid on dish.

Allow plates to dry on bench. Once the plates are dry, place them in the plastic sleeves and store at 4°C. Allow 24-48 hours for media to fully reduce if using for anaerobic work.

***C. innocuum* isolation**

This study was approved by Clemson University’s Institutional Review Board. Healthy donors were over 18, had not taken antibiotics or been diagnosed with any infections within six months, and were not immunocompromised or diagnosed with chronic gastrointestinal conditions. The only information collected from individuals were age and sex. Following informed consent, donors were asked to collect a fecal sample using a commode specimen collection kit (Fisherbrand, Cat. #02-544-208) and a collection tube (Sarstedt Inc, Cat. #80.734.311) and return the sample within the same day, storing on ice if necessary. Upon receipt, the fecal sample was passed into an anaerobic chamber (Coy Laboratory Products, Grass Lake, MI) to generate 0.5 g aliquots for microbial isolation described here under anaerobic conditions (85% nitrogen, 10% hydrogen, 5% carbon dioxide) and storage at −80°C for future use. Three methods of streaking were applied from the fecal samples: 1) material directly onto agar plates (stool streak), 2) a fecal slurry adding 10 μL to 1 mL of sterile, pre-reduced phosphate buffer (PBS) (fecal slurry) to streak on to agar plates, and 3) inoculation into broth growth for 24 hours before plating onto specific media using dilutions (dilution). We used the following media to isolate *C. innocuum*: Brain Heart Infusion (BHI) (1), BHI supplemented with fetal bovine serum (FBS; 50 mL/L BHI), or Taurocholate Cycloserine-Cefoxitin-Fructose (TCCFA) (2, 3). For direct streaking or fecal slurry, 10 μL loop of material was streaked onto each agar media type and incubated at 37°C for at least 24 hours. For broth inoculation, 10 μL of fecal slurry was added to 5 mL of media and incubated at 37°C for 24 hours. 20 μL of broth was then serially diluted up to 10^-6^, plated on agar plates of the corresponding media, and further incubated for up to 96 hours. Single colonies were picked and streaked for purity. Once the isolates were visibly pure, they were inoculated into 2 mL of their corresponding broth for overnight growth at 37°C. The broth growth for each isolate was split into three aliquots: a 20% glycerol stock stored at −80°C for future *in vitro* characterization, diluted 10^-1^ for heat extraction for PCR (65°C for 20 minutes, then stored at −20°C), and the remaining broth was stored at −20°C for DNA extraction.

**Bromocresol purple (BCP) assay**. The 96-well plate used for growth assessment was removed and 2 μl of bromocresol purple (BCP, 1:10 dilution) was added to all wells and gently mixed, then placed into a Synergy H1 Hybrid Reader and OD_588_ was measured. To assess BCP absorbance and pH correlation of different media, BMCA and positive control media TCCFB were titrated to pH values 4.2-8.4 in increments of 0.2. Upon titration, 2 μl of BCP (1:10 dilution) was added to a 96-well plate containing 198 μL of titrated media and gently mixed. The OD_588_ was measured, and a linear regression (y= −0.73 + 0.18x, R^2^ = 0.94) was fit to the data (Supplementary Figure S4). Using this regression, expected absorbance values were calculated based on actual pH values. Expected values were then checked against actual absorbance values to verify the assay. All *in vitro* growth experiments were performed in triplicate for each sugar type and strain. Growth curve data are displayed as mean values ± standard error for pooled data for each group. Statistical comparisons for growth were performed via one-way analysis of variance (ANOVA) on sugar types per strain using area-under-curve (AUC) analysis. ANOVA was followed by Tukey’s HSD with a P-value *, < 0.05; **, < 0.005: ***, < 0.0001 considered significant.

**Whole genome assembly and phylogeny through maximum likelihood trees**. All data processing was conducted using a bash script on the Clemson University High Performance Supercomputer, Palmetto (available at: <https://github.com/SeekatzLab/C.innocuum-diversity>). The raw reads were quality-checked and adapter-trimmed using Trim-galore (4) then assembled using SPAdes (5) with –careful flag and chosen k-mers of 55, 77 and 127, as optimized using MEGAHIT (6). Quast with MultiQC was used to calculate assembly statistics (Supplementary Table S1) (7, 8). Average coverage was calculated using Bowtie2 and SAMtools (9, 10). Prokka was used to annotate assemblies (11). To verify the assembly identity, annotations were run through NCBI Blast and EzBioCloud. Assemblies were also mapped on to the Genome Taxonomy Database (GTDB) (12) through GTDB-tk using Peptostreptococcaceae (i.e., containing *Clostridioides difficile*) as the taxon outgroup (13). After verification, additional sequences that belonged to *C. innocuum* and its two neighboring genera were obtained for making maximum likelihood trees from GTDB, Cherny et al, 2022, and Ha, et al, 2020 (Supplementary Table S1) (14, 15).

Maximum likelihood trees from the *C. innocuum* core genome SNP sites was determined by Roary and converted to phylip-format (16, 17) using RAxML 8.2.12 (18) by bootstrapping 500 times. The 16S rRNA maximum likelihood tree was aligned using Clustal Omega and bootstrapped 500 times by RAxML (18, 19). Another maximum likelihood phylogenetic tree was created using amino acid fasta sequences mapped against the “phylophlan” database with DIAMOND in Phylophlan (20). Trees were visualized either using Graphlan (21) or RStudio 1.4.1106 with ggtree and treeio packages (22, 23). All bioinformatic tools and R packages have been listed in Supplementary Table S4.

**Functional enrichment and pangenome analysis**. Contigs from SPAdes were reformatted, and annotated with the COG and KEGG database using Anvi’o version 7.0 (24). Anvi’o was also used to create the pangenome by creating a genome storage. For functional enrichment, the pangenome was equipped by a user defined category text file classifying the strains in the clades identified from the trees using anvi-import-misc-data. The functional enrichment was defined using the category variable as the clades and annotation source as KEGG Modules, which uses a Generalized Linear Model with the logit linkage function to generate an enrichment score and p-value for each function. False Detection Rate correction to p-values was applied using the package qvalue from Bioconductor (25). The functional enrichment was visualized in RStudio using dplyr, ggplot2 and readxl packages (26-28). The pangenome was visualized using anvi-display-pan from Anvi’o. Heap’s law was calculated in Rstudio (formulated as n = κN^γ^, where n is the pan-genome size, N is the number of genomes used, and κ and γ are the fitting parameters) and the α parameter was calculated using micropan (29, 30).

**Average nucleotide identity (ANI) and dereplication**. Average nucleotide identity (ANI) was computed using the anvi-compute-genome-similarity that used pyANI (31) for calculating the similarity between the strains using Anvi’o. Dereplication between strains was computed using pyANI in anvi-dereplicate-genomes. The dereplication was calculated at 90, 95, 98, 99, 99.9 and 100 % similarity threshold, with 95, 99, 100%. Ten strains from the entire set depreplicated at 100%, indicating they were the same strains.

**CAZyme analysis**. CAZymes were predicted using DBCAN version 2.0.6 (32), which assigns CAZy family and subfamily using similarities to the hidden markov models (HMM) of the CAZy proteins. DBCAN was run with the Fasta nucleotide sequences generated from Prokka for each of the strains. The resultant overview files were used to analyze CAZymes in RStudio.

**Virulence factor, toxin and antimicrobial peptides resistance analysis.** Prokka generated nucleotide fasta files (.fna) were processed through PathoFact (33) to predict virulence factors, toxins, and antimicrobial peptides. Genomic islands were predicted in *C. innocuum* 14501 using the web computational tool IslandViewer 4 (34). The prediction is made either according to hidden Markov models and measuring codon usage or % GC and presence of mobility genes. Circos was used to visualize genomic islands (34).

**References**

1. Sorg JA, Dineen SS. 2009. Laboratory Maintenance of Clostridium difficile. Current Protocols in Microbiology 12:9A.1.1-9A.1.10.

2. George WL, Sutter VL, Citron D, Finegold SM. 1979. Selective and Differential Medium for Isolation of *Clostridium difficile*. J Clin Microbiol 9:214-219.

3. Wilson KH, Silva J, Fekety FR. 1981. Suppression of Clostridium difficile by Normal Hamster Cecal Flora and Prevention of Antibiotic-Associated Cecitis. Infection and Immunity 34:626-628.

4. Martin M. 2011. Cutadapt removes adapter sequences from high-throughput sequencing reads. 2011 17:3.

5. Prjibelski A, Antipov D, Meleshko D, Lapidus A, Korobeynikov A. 2020. Using SPAdes De Novo Assembler. Current Protocols in Bioinformatics 70:e102.

6. Li D, Liu CM, Luo R, Sadakane K, Lam TW. 2015. MEGAHIT: an ultra-fast single-node solution for large and complex metagenomics assembly via succinct de Bruijn graph. Bioinformatics 31:1674-6.

7. Mikheenko A, Prjibelski A, Saveliev V, Antipov D, Gurevich A. 2018. Versatile genome assembly evaluation with QUAST-LG. Bioinformatics 34:i142-i150.

8. Ewels P, Magnusson M, Lundin S, Käller M. 2016. MultiQC: summarize analysis results for multiple tools and samples in a single report. Bioinformatics 32:3047-3048.

9. Langmead B, Wilks C, Antonescu V, Charles R. 2019. Scaling read aligners to hundreds of threads on general-purpose processors. Bioinformatics 35:421-432.

10. Danecek P, Bonfield JK, Liddle J, Marshall J, Ohan V, Pollard MO, Whitwham A, Keane T, McCarthy SA, Davies RM, Li H. 2021. Twelve years of SAMtools and BCFtools. Gigascience 10.

11. Seemann T. 2014. Prokka: rapid prokaryotic genome annotation. Bioinformatics 30:2068-9.

12. Parks DH, Chuvochina M, Rinke C, Mussig AJ, Chaumeil PA, Hugenholtz P. 2022. GTDB: an ongoing census of bacterial and archaeal diversity through a phylogenetically consistent, rank normalized and complete genome-based taxonomy. Nucleic Acids Res 50:D785-D794.

13. Chaumeil PA, Mussig AJ, Hugenholtz P, Parks DH. 2019. GTDB-Tk: a toolkit to classify genomes with the Genome Taxonomy Database. Bioinformatics doi:10.1093/bioinformatics/btz848.

14. Ha CWY, Martin A, Sepich-Poore GD, Shi B, Wang Y, Gouin K, Humphrey G, Sanders K, Ratnayake Y, Chan KSL, Hendrick G, Caldera JR, Arias C, Moskowitz JE, Ho Sui SJ, Yang S, Underhill D, Brady MJ, Knott S, Kaihara K, Steinbaugh MJ, Li H, McGovern DPB, Knight R, Fleshner P, Devkota S. 2020. Translocation of Viable Gut Microbiota to Mesenteric Adipose Drives Formation of Creeping Fat in Humans. Cell 183:666-683 e17.

15. Cherny KE, Muscat EB, Balaji A, Mukherjee J, Ozer EA, Angarone MP, Hauser AR, Sichel JS, Amponsah E, Kociolek LK. 2022. Association Between Clostridium innocuum and Antibiotic-Associated Diarrhea in Adults and Children: A Cross-sectional Study and Comparative Genomics Analysis. Clinical Infectious Diseases doi:10.1093/cid/ciac483.

16. Page AJ, Taylor B, Delaney AJ, Soares J, Seemann T, Keane JA, Harris SR. 2016. SNP-sites: rapid efficient extraction of SNPs from multi-FASTA alignments. Microb Genom 2:e000056.

17. Page AJ, Cummins CA, Hunt M, Wong VK, Reuter S, Holden MT, Fookes M, Falush D, Keane JA, Parkhill J. 2015. Roary: rapid large-scale prokaryote pan genome analysis. Bioinformatics 31:3691-3.

18. Stamatakis A. 2014. RAxML version 8: a tool for phylogenetic analysis and post-analysis of large phylogenies. Bioinformatics 30:1312-3.

19. Sievers F, Higgins DG. 2018. Clustal Omega for making accurate alignments of many protein sequences. Protein Sci 27:135-145.

20. Asnicar F, Thomas AM, Beghini F, Mengoni C, Manara S, Manghi P, Zhu Q, Bolzan M, Cumbo F, May U, Sanders JG, Zolfo M, Kopylova E, Pasolli E, Knight R, Mirarab S, Huttenhower C, Segata N. 2020. Precise phylogenetic analysis of microbial isolates and genomes from metagenomes using PhyloPhlAn 3.0. Nat Commun 11:2500.

21. Asnicar F, Weingart G, Tickle TL, Huttenhower C, Segata N. 2015. Compact graphical representation of phylogenetic data and metadata with GraPhlAn. PeerJ 3:e1029.

22. Yu G. 2020. Using ggtree to Visualize Data on Tree-Like Structures. Current Protocols in Bioinformatics 69:e96.

23. Wang LG, Lam TT, Xu S, Dai Z, Zhou L, Feng T, Guo P, Dunn CW, Jones BR, Bradley T, Zhu H, Guan Y, Jiang Y, Yu G. 2020. Treeio: An R Package for Phylogenetic Tree Input and Output with Richly Annotated and Associated Data. Mol Biol Evol 37:599-603.

24. Eren AM, Esen OC, Quince C, Vineis JH, Morrison HG, Sogin ML, Delmont TO. 2015. Anvi'o: an advanced analysis and visualization platform for 'omics data. PeerJ 3:e1319.

25. Shaiber A, Willis AD, Delmont TO, Roux S, Chen LX, Schmid AC, Yousef M, Watson AR, Lolans K, Esen OC, Lee STM, Downey N, Morrison HG, Dewhirst FE, Mark Welch JL, Eren AM. 2020. Functional and genetic markers of niche partitioning among enigmatic members of the human oral microbiome. Genome Biol 21:292.

26. Wickham H. 2016. ggplot2: Elegant Graphics for Data Analysis, Springer-Verlag New York. <https://ggplot2.tidyverse.org>.

27. Wickham H, François R, Henry L, Müller K. 2022. dplyr: A Grammar of Data Manipulation., <https://dplyr.tidyverse.org>, <https://github.com/tidyverse/dplyr>.

28. Wickham H, Bryan J. 2022. readxl: Read Excel Files., <https://readxl.tidyverse.org>, <https://github.com/tidyverse/readxl>.

29. Snipen L, Liland KH. 2015. micropan: an R-package for microbial pan-genomics. BMC Bioinformatics 16:79.

30. Tettelin H, Riley D, Cattuto C, Medini D. 2008. Comparative genomics: the bacterial pan-genome. Curr Opin Microbiol 11:472-7.

31. Pritchard L, Glover RH, Humphris S, Elphinstone JG, Toth IK. 2016. Genomics and taxonomy in diagnostics for food security: soft-rotting enterobacterial plant pathogens. Analytical Methods 8:12-24.

32. Zhang H, Yohe T, Huang L, Entwistle S, Wu P, Yang Z, Busk PK, Xu Y, Yin Y. 2018. dbCAN2: a meta server for automated carbohydrate-active enzyme annotation. Nucleic Acids Res 46:W95-W101.

33. de Nies L, Lopes S, Busi SB, Galata V, Heintz-Buschart A, Laczny CC, May P, Wilmes P. 2021. PathoFact: a pipeline for the prediction of virulence factors and antimicrobial resistance genes in metagenomic data. Microbiome 9:49.

34. Bertelli C, Laird MR, Williams KP, Simon Fraser University Research Computing G, Lau BY, Hoad G, Winsor GL, Brinkman FSL. 2017. IslandViewer 4: expanded prediction of genomic islands for larger-scale datasets. Nucleic Acids Res 45:W30-W35.
